# The Synergistic Antitumor Effect of Tanshinone IIA Plus Adriamycin on Human Hepatocellular Carcinoma Xenograft in BALB/C Nude Mice and Their Influences on Cytochrome P450 CYP3A4 *In Vivo*

**DOI:** 10.1155/2020/6231751

**Published:** 2020-02-29

**Authors:** Tao-Li Liu, Li-Na Zhang, Yue-Yu Gu, Mei-Gui Lin, Jun Xie, Yu-ling Chen, Jia-Hui Liu, Xin-Lin Wu, Sui-Lin Mo

**Affiliations:** ^1^The Seventh Affiliated Hospital, Sun Yat-Sen University, Shenzhen 518107, China; ^2^The Sixth Affiliated Hospital, Sun Yat-Sen University, Guangzhou 510655, China; ^3^The Second Clinical College, Guangzhou University of Chinese Medicine and Guangdong Provincial Academy of Chinese Medical Sciences, Guangzhou 510080, China; ^4^Li Wan District Shi Wei Tang Street Community Health Service Center, Guangzhou 510360, China; ^5^Peking Union Medical College Hospital, Peking Union Medical College, China Academy of Medical Sciences, Beijing 100730, China; ^6^Sydney Acupuncture & Chinese Medicine Centre, New South Wales 2220, Australia; ^7^The First Affiliated Hospital, Sun Yat-Sen University, Guangzhou 510080, China

## Abstract

**Objective:**

Hepatocellular carcinoma is one of the most common diseases that seriously threaten human life and health. In this study, we evaluated the inhibitory effect of tanshinone IIA (Tan IIA) combined with adriamycin (ADM) on human hepatocellular carcinoma and developed a platform to assess the function if Chinese herbal ingredients combined with chemotherapy drugs have synergistic antitumor effects *in vivo*.

**Methods:**

Established animal model of human hepatocarcinoma HepG2 cell in nude mice. Mice were divided into model control group, Tan IIA group, ADM group, and Tan IIA + ADM group. The changes from general condition, weight, tumor volume, and inhibition rate were observed. The data were gathered from serum AST level and histopathological changes. The content and activity of cytochrome P450 were determined by spectrophotometric analysis. CYP3A4 protein expression was analyzed by western blotting. The binding model crystal structure of Tan IIA and ADM with pregnane X receptor (PXR) was evaluated by Discovery Studio 2.1.

**Results:**

A combination of Tan IIA with ADM could improve life quality by relieving ADM toxicity, decreasing tumor volume, declining serum AST level, and improving liner pathological section in tumor-bearing mice. The inhibitory rates of Tan IIA, ADM, and cotreatment were 32.77%, 60.96%, and 73.18%, respectively. The Tan IIA group significantly enhanced the content of cytochrome b5, P450, and erythromycin-*N*-demethylase activity. CYP3A4 protein expression was enhanced obviously by the Tan IIA + ADM group. Virtual molecular docking showed that both Tan IIA and ADM could be stably docked with the same binding site of PXR but different interactions.

**Conclusions:**

Tan IIA in combination with ADM could improve the life quality in tumor-bearing mice and enhance the antitumor effect. The Tan IIA group increased the concentration of cytochrome P450 enzymes and activity. Combined Tan IIA with ADM could upregulate the CYP3A4 protein expression and make relevant interaction with protein PXR by virtual docking.

## 1. Introduction

Hepatocellular carcinoma (HCC) is one of the most malignant of human tumor types, characterized by high mortality rates and aggressive metastatic potential [1, 2]. Surgical resection is the first-line therapy for HCC; however, systemic chemotherapy plays an integral role for patients with advanced HCC for whom surgery is not a feasible option [3]. Nevertheless, the chemotherapeutic drugs have lots of side effects, such as myelosuppression, gastrointestinal tract toxicity, and immune system disorder. So preventing HCC therapies is a significant issue. Moreover, drug resistance also substantially limits the advancement of chemotherapy in HCC [4]. Many herbal medicines have proved to contain numerous anticancer components, but their anticancer activity was found to be inferior to antineoplastic drugs when herbal medicines were used alone [5–7].

Currently, more and more herbs are regarded as supplementary medicines when combined with antitumor medicine in many therapeutic areas [8, 9]. Our preliminary work [10, 11] had shown that Chinese medicine herbs may have the advantage of enhancing drug efficacy and reducing toxicity during chemotherapy. Adriamycin (ADM) is a well-known chemotherapeutic drug in the treatment of various cancers. However, ADM has many side effects, including bone marrow depression, cardiac toxicity, hematological system damage, and other adverse reactions. Additionally, the emergence of drug resistance and these potential side effects highlight the major limitations of using ADM alone in its current clinical application [12, 13].

Tanshinone IIA (Tan IIA: 1,6,6-trimethyl-6,7,8,9-tetrahydrophenanthro[1,2-b] furan-10,11-dione) is the major antitumor bioactive component of phenanthrenequinone isolated from “Dan-Shen” which is one of the most popular Chinese herbs used in China. Recent studies have shown that Tan IIA can suppress tumor cell proliferation, induce apoptosis and cell cycle arrest, disturb tumor cell cycle, and inhibit tumor cell invasion or transfer [10, 14–17]. Some researchers have reported that Tan IIA can also protect doxorubicin-induced apoptosis [18, 19]. Unfortunately, like other antitumor active components from herbs, Tan IIA was confirmed to have weaker antitumor activity compared with currently used chemotherapeutics. In our preliminary work, Xie et al. [10] found that both Tan IIA and ADM had antiproliferative effects on A549 cells, but ADM had a stronger antitumor effect than Tan IIA. In the following study, Xie et al. [13] also found that Tan IIA and ADM inhibited the proliferation of NSCLC A549, PC9, and HLF cells in a time- and dose-dependent manner, while the proliferative inhibition effect of Tan IIA on cells was much weaker than that of ADM.

To investigate the interactions between the antitumor active components from herbal medicines and chemotherapeutic medicines, our research had already built a platform that combined *in vitro* laboratory experiments with virtual laboratory work in previous studies [10, 11, 13, 20]. In this study, we further develop this platform based on *in vivo* studies and virtual computing for Tan IIA, which had proved to have a demonstrably antitumor effect previously. Meanwhile, ADM, a classic antitumor drug, was chosen as the main object of this study. To explore the antitumor mechanisms and interactions between Chinese herbs and chemotherapy medicines, the effect of Tan IIA in combination with ADM on tumor-bearing animals, drug-metabolizing enzymes, protein expressions, and drug virtual screening were tested.

## 2. Materials and Methods

### 2.1. Cell Line, Cell Culture, and Reagents

The human liver hepatocellular carcinoma HepG2 cell was purchased from the Cell Bank of the Chinese Academy of Sciences (CAS, Shanghai, China) and maintained in DMEM (Gibco, New York, USA) supplemented with 10% fetal bovine serum (Utah, Hyclone, USA) in a humidified incubator at 37°C, 5% CO_2_ atmosphere. Tan IIA (sulfotanshinone sodium injection, 5 mg/mL) was commercially available from the Biochemical Pharmaceutical Co., Ltd., Shanghai, China. Doxorubicin hydrochloride injection was supplied by Main Luck Pharmaceuticals, Shenzhen, China. Lyophilized powder with the purity 99.99%, as Na_2_S_2_O_4_ (Thermo, Waltham, USA), erythromycin and NADPH (Solarbio, Beijing, China), and Ba(OH)_2_ (Sigma-Aldrich, St. Louis, USA) were used in the study. 99.99% carbon monoxide gas was obtained from Yue Du, Guangzhou, China. All chemicals were of analytical grade and were of the highest purity available.

### 2.2. Animal Model and Experimental Protocols

A total of twenty male BALB/c nude mice at 4–6 weeks of age were purchased from the Experimental Animal Center of Guangdong Province, Guangzhou, China (the Animal Certificate No. 44007200004290), and raised in a specific pathogen-free (SPF) room under standardized housing conditions (temperature 25–27°C, relative humidity 45–50%). The *in vivo* study protocol was approved by the Animal Ethics Committees of the First Affiliated Hospital, Sun Yat-sen University, Guangzhou, China, and all experiments were conducted in compliance with relevant regulations and ethics codes. The experiments were performed with care to avoid stress to the mice.

**Figure 1:**
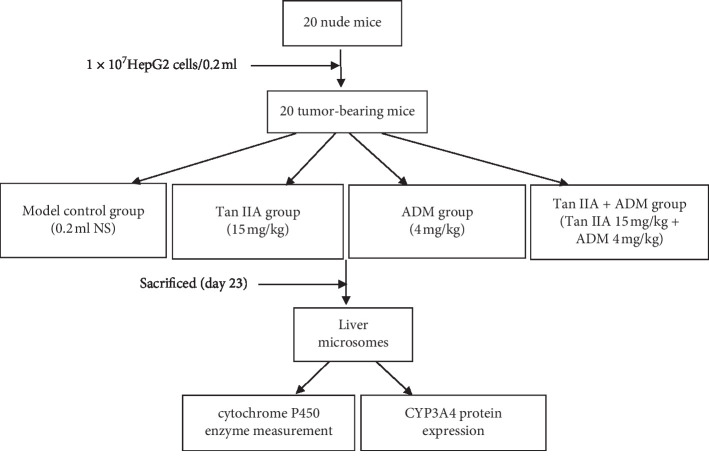
Experimental design of the xenograft animal model. HepG2 cell tumor xenograft in BALB/c nude mice. Mice were divided randomly into four groups: model control group, Tan-IIA (15 mg/kg of body weight, day 1∼7, day 17∼23), ADM (4 mg/kg of body weight, day 1∼3, day 17∼19), and Tan IIA + ADM group (Tan-IIA 15 mg/kg plus ADM 4 mg/kg).

HepG2 cells were collected in the exponential phase and digested into single-cell suspensions. After housing for 1 week, nude mice were inoculated subcutaneously with 1 × 10^7^ cells in 0.2 mL phosphate-buffered saline at the right dorsal proximal upper limbs. The mice were disinfected and placed in a laminar air flow rack while their physical signs were monitored. When tumor sizes reached 100 mm^3^ in volume, the tumor-bearing mice were randomly divided into four groups: model control group (0.2 mL of saline, day 1∼7, day 17∼23), Tan IIA group (15 mg/kg of body weight, day 1∼7, day 17∼23), ADM group (4 mg/kg of body weight, day 1∼3, day 17∼19), and cotreatment group (Tan IIA 15 mg/kg plus ADM 4 mg/kg). Each agent was injected intravenously by tail. A schematic of the experimental design is shown in Figure 1.

During the entire process, mice were weighed and tumor sizes (length and width) were measured once every 5 days. On day 23, mice were sacrificed, tumors were completely stripped, and livers of mice were dissected for further examination. Tumor size (tumor weight and tumor volume) was calculated at the end of experiment using the following formula [21]: tumor volume (*V*, mm^3^) = 0.5 × length × width [2]. The tumor growth inhibition rate was calculated in our study as follows: inhibition rate (IR, %) = (mean tumor weight of the model group—mean tumor weight of the treatment group)/mean tumor weight of the model group × 100%.

### 2.3. Preparation of Mouse Hepatic Microsomes

On day 23, mice were sacrificed and livers were collected and transferred to ice immediately after surgical excision. The livers were cut into pieces at 4°C and stored at −80°C until the microsomes were prepared. The liver microsomes were prepared by differential centrifugation as described previously [22]. Liver samples were thawed and weighed, and 3 volumes of ice-cold homogenization medium (50 mM Tris-HCl buffer at pH 7.4 containing 0.25 M sucrose at 4°C) were then added. The tissue was chopped using scissors and fully homogenized via a glass pestle for 30 min. The resultant homogenates were transferred to clean centrifuge tubes and centrifuged at 15000 g for 20 min at 4°C using a Beckman centrifuge (Beckman Coulter, Inc., Fullerton, CA, USA). The supernatant was collected and centrifuged at 100,000 g for 60 min at 4°C using an Optimal L-100 XP Beckman ultracentrifuge. The microsomal pellet was resuspended with a homogenization medium. Hepatic microsomal suspensions were liquefied (1 mL) into a 1.5 mL test tube and stored at 80°C until used. Protein concentrations of microsomes were determined using the Lowry method [23].

### 2.4. Spectrophotometric Analysis

Reduced carbon monoxide (CO) difference spectra were recorded on a Hitachi (Danbury, CT) U3300 dual-beam spectrophotometer, and data were analyzed using UV Solutions 1.2 software (Hitachi). In brief, 0.3 mL incubation (containing 1 g/mL liver microsome) in 5.7 mL of 0.1 M potassium phosphate buffer (pH 7.4) was prepared, and after a 2 min incubation at 37°C, aliquots were reduced by addition of 40 *μ*L of 10% sodium dithionite and then evenly divided between two 1 mL, 1 cm path length cuvettes. A baseline was recorded, and then carbon monoxide was gently bubbled into the sample cuvette (approximately 1 bubble per sec for 1 minute). Then, absorbance was scanned from 400 to 500 nm, and peak absorbance at 450 nm and 490 nm was determined.

### 2.5. Measurement of Enzymatic Activity in Liver Microsomes

To determine the enzymatic activities for CYP3A in mice hepatic microsomes, the activities of CYP3A-related erythromycin *N*-demethylase (ERND) were assayed by spectrophotometric analysis at 37°C. The ERND enzymatic activity was determined by measuring the formation of formaldehyde. To analyze ERND activity, 0.1 mL of 1 g/mL liver microsomes, 1.7 mL of 0.1 mol/L phosphate buffer saline (PBS), and 0.1 mL of 4 mmol/L erythromycin were mixed in 0.1 mL of 10 mmol/L NADPH to reach a total volume of 2 mL. After 30 min at 37°C, the reaction was stopped by the addition of 1 mL methanol. After centrifugation at 5000 g for 10 min, the reaction product resorufin in the supernate was measured fluorometrically. The ERND activity was determined by testing the formation of formaldehyde at 420 nm, based on a previous study [24, 25].

### 2.6. Western Blotting Analysis

The level of CYP3A4 activity in mice liver microsomes was determined by western blotting. Protein samples were mixed with equal volumes of 5 × SDS-PAGE loading buffer and separated using an SDS-PAGE gel after heating at 95°C for 4 min and then were transferred to PVDF membranes (Millipore, Bedford, MA, USA). The membranes were blocked with 5% skim milk in Tween 20 (0.05%)-PBS (pH 7.4) for 2 h at room temperature and incubated overnight at 4°C with rabbit CYP3A4 polyclonal antibodies (1 : 800, ABclonal, Boston, USA). Next, the membrane was washed three times with TBST buffer and incubated with horseradish peroxidase- (HRP-) conjugated secondary antibodies (1 : 1000; Cell Signaling Technology, Danvers, MA, USA) for 2 h at room temperature and washed extensively before detection. The membranes were developed using enhanced chemiluminescence (Millipore, Bedford, MA, USA), and membranes were exposed to Kodak XAR-5 films (Sigma-Aldrich). GAPDH (1 : 1000, Cell Signaling Technology, Danvers, MA, USA) was used as the internal reference. Relative optical density (ratio to GAPDH) of each blot band was quantified by using National Institutes of Health (NIH) image software (Image J1.36b).

### 2.7. Molecular Docking Study

To predict the potential protein-ligand interactions between tested drugs (Tan IIA, and ADM) and targeted protein (pregnane X receptor, PXR), after accuracy testing, two modules (CDOCKER and LibDock) in Discovery Studio (DS) 2.1 (Accelrys Software Inc., San Diego, USA) were applied to the molecular docking algorithm in this study. The calculation of root mean square deviation (RMSD) was carried out for the validation of the veracity for the selection of molecular docking modules in DS 2.1. The three-dimensional (3D) crystal structure of PXR was selected from PDB (http://www.rcsb.org/pdb/) with an ID of 4NY9. The 3D structures of Tan IIA and ADM were downloaded from the PubChem Project (http://www.pubchem.ncbi.nlm.nih.gov/) with CIDs of 164676 and 123097261, respectively. The DS 2.1 program was run on a localhost 9943 server. The basic docking procedures were performed as follows.

First, water molecules in the protein were removed and hydrogen atoms were added to the protein. Small molecules and selected proteins were refined with CHARMM. Second, we defined the active site of PXR by two methods, one according to internal ligand's binding site while the second was defined automatically with DS 2.1. Third, after refined with CHARMM, Tan IIA and ADM were docked into the active site of 4NY9 (the PXR protein's PDBID) which was carried out by consideration of electrostatic energy and van der Waals (vdW) force.

Docking procedures for CDOCKER are as follows: the grid origin was located at the center of the active site of PXR, with the smallest of 21.0 Å or the largest ligand dimension +5.0 Å as the side length. The grid spacing was 0.5 Å. Each vdW or electrostatic probe was positioned at these grid points. A trilinear interpolation was used to evaluate the ligand atoms' energies between grid points. A harmonic potential with the force constant of 300 kcal/mol was applied outside the grid boundary. Using Tan IIA and ADM configuration of new ligands, it randomly generated a set of ten different orientations. These orientations docked into the PXR protein (4NY9) and moved into the center of the grid by performing a series of random rotations. Once the randomized ligand was docked in the active site, an MD simulation was run consisting of a heating phase from 300 to 700 K with 2,000 steps, followed by a cooling phase back to 300 K (5,000 steps). The energy threshold for vdW force was set at 300 K. Finally, a short energy minimization used to refine the simulation docking results when MD ended consisted of 50 steps of steep descent followed by up to 200 steps of conjugate gradient using an energy tolerance of 0.001 kcal/mol as described previously [13, 20].

### 2.8. Statistical Analysis

Data were presented as mean± standard deviation (SD) and analyzed using SPSS 13.0 software. Differences between groups of continuous variables were evaluated by ANOVA analysis. The control and experimental animal groups were analyzed using an unpaired Student's *t*-test. A *P* value of 0.05 or less was considered significant.

## 3. Results

### 3.1. Changes of General Condition and Weight in HepG2 Tumor-Bearing Mice

**Figure 2:**
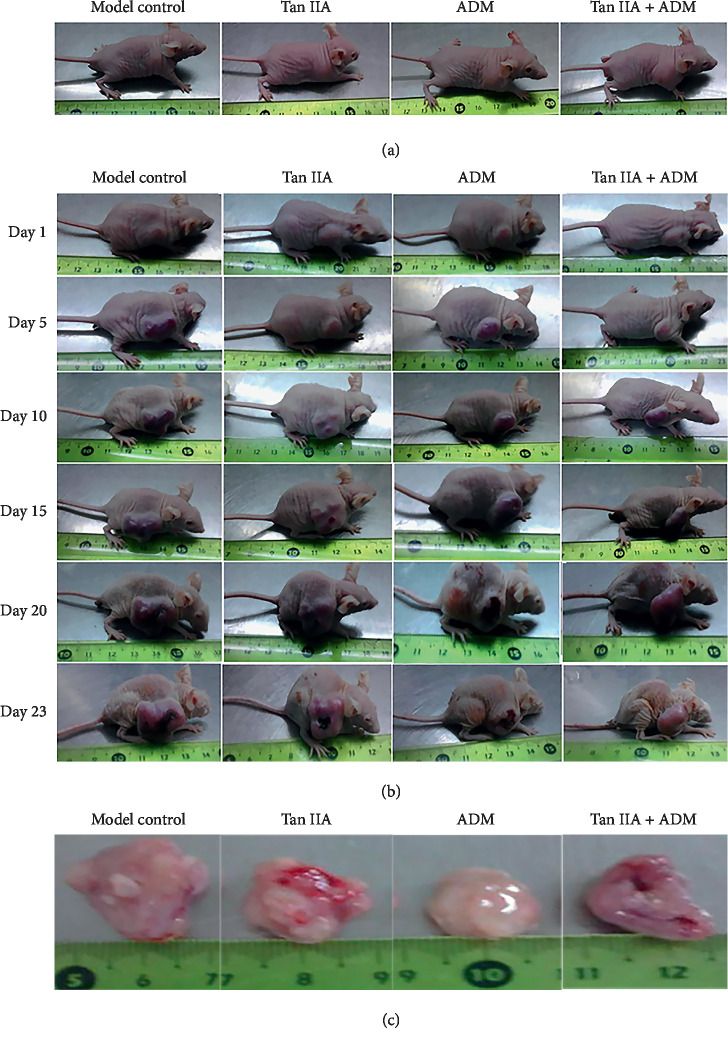
Changes of general condition and tumor growth in HepG2 tumor-bearing mice. (a) Tumor-bearing nude mice before treatments. (b) The changes of general condition and weight growth during treatments in tumor-bearing nude mice. (c) The photograph shows the representative mice tumors in the different treatment groups.

At the beginning, the general condition of all mice was well, no death or anorexia, more that, they gained weight steadily, kept shiny coat, with better appetite, normal activity, and excretion. However, on the 15th day, the weight of mice increased slowly. Four groups showed differences: mice in the model control group and Tan IIA group remained almost in normal state, with lively behavior and increased diet. While ADM and Tan IIA + ADM group showed poor condition, with lower spirit, worse appetite, liquid stools, and a little dull activity. Among them, the ADM group was one of the worst states which included worst dull skin, huddled up and dispirited. The Tan IIA plus ADM group reflected a better ordinary state than the ADM group but also had lighter weight. On the 20th day, a cachexia state occurred in ADM and Tan IIA + ADM groups, with visibly emaciation, lethargy, photophobia, photophobia, timidity, and even having ulcerated and ruptured tumor tissues (Figure 2).

**Figure 3:**
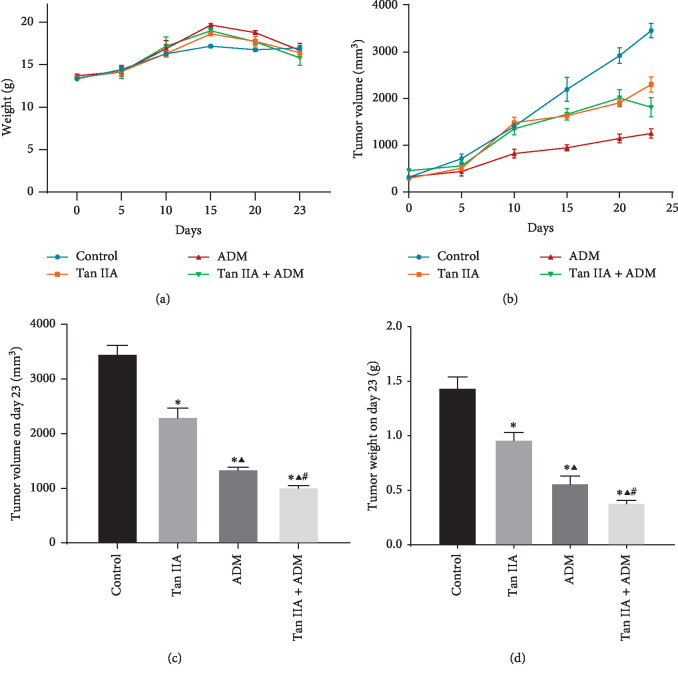
Effects of Tan IIA or AMD treatments on the HepG2 tumor-bearing mice. (a) Body weight in different treatment groups. (b) Tumor volume of mice was recorded during treatments. (c, d) Effects of different treatments on final tumor volume and weight when compared on the 23th day. *N* = 5 except for the Figures 3(c) and 3(d) in the ADM group (*n* = 4) and the Tan IIA + ADM group (*n* = 3) ^*∗*^*P* < 0.01 versus control group; ^*▲*^*P* < 0.01 versus Tan IIA group; ^#^*P* < 0.01 versus ADM group.

During the first fifteen days, mice grew up quickly, with 0.22 g–0.36 g/day. Interestingly, after the 15th day, mice's weight began to slow down in all groups. On the 23th day, the weight of experimental animals was increased by over 10%. Compared with the model control group, the weight of the ADM group and Tan IIA + ADM group presented obvious change and with statistical differences (*P*=0.017), but there were no differences between *W*_0_ and *W*_23_ in other groups (*P* > 0.05). Based on the relative formula, the rate of weight increase in the model control group was the highest by 27.24%, the Tan IIA group and ADM group had lower proportion, with 22.12% and 21.78%, respectively. The Tan IIA plus ADM group tended to be better off, with the lowest levels for 17.30% (Figure 3(a)).

### 3.2. Change of Tumor Volume, Tumor Weight, the Inhibitory Rate, and the *Q* Value in Tumor-Bearing Mice

The results indicated that (Figures 3(b)–3(d)) a tumor in the treatment group grew fast at first, but slower than in the model control group. After the 15th day, the tumor in Tan IIA plus ADM group grew more slowly than in other groups. On days 20 and 23, some tumor tissues were scratched by mice themselves; the phenomenon was observed that the tumor tissues became ulcerated and ruptured in the ADM group (one mouse sample) and the Tan IIA + ADM group (two mice samples), particularly in the Tan IIA + ADM group, so Figure 3(b) shows a sharp decline between days 20–23. So, we speculated that tumor volume became smaller which might be related to the ulcerated tumor tissues in nude mice. Tumor-ulcerated mice samples were removed in the ADM and combined group when the statistical data were analyzed (Figures 3(b)–3(d)).

The results showed that the tumor volume size in treatment groups was significantly reduced compared to the model control group, with a statistical significance (*P* < 0.001). The Tan IIA + ADM group was the smallest. Meanwhile, the tumor weight in the Tan IIA + ADM group was the lightest. Compared to the model control group, the inhibitory rate in the Tan IIA group was 32.77%; the second lowest rate was in the ADM group (60.96%), and finally, the Tan IIA + ADM group had the lowest rate (73.18%) as shown in Table 1. According to the Jin's modified Bürgi's formula, we calculate the *Q* value which was about 0.988 to evaluate the combined effect of drugs.

### 3.3. Determination of Serum AST Level in HepG2 Tumor-Bearing Nude Mice

**Figure 4:**
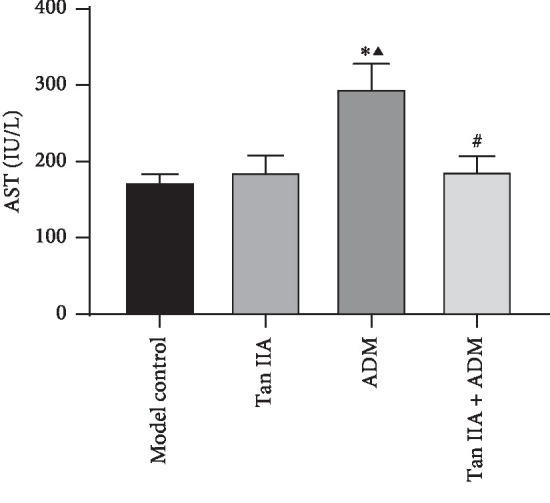
Effect of Tan IIA and ADM on the serum AST level in HepG2 tumor-bearing nude mice (*n* = 5, ^*∗*^*P* < 0.01 versus control group; ^*▲*^*P* < 0.01 versus Tan IIA group; ^#^*P* < 0.01 versus ADM group).

Mice blood samples were obtained from the abdominal aorta at the end of experiment and the serum AST level was examined. The histological abnormalities have coincided with increased activity of AST (Figure 4) Each group of AST level had risen inordinately and could induce mice hepatotoxicity. In model control mice, serum AST level was relatively low, but the level of the ADM group increased obviously, which could lead to approximately one-third enhancement in the value of AST compared to the model control group, Tan IIA group, and Tan IIA + ADM group separately. Administration of Tan IIA to mice resulted in some degree reduction in AST; the combination of Tan IIA and ADM treatment could decrease AST average level to about 6.75% than the Tan IIA group, and there was no statistically significant difference.

### 3.4. Tan IIA Ameliorated ADM-Induced Histopathological Changes in the Liver of HepG2 Tumor-Bearing Mice

**Figure 5:**
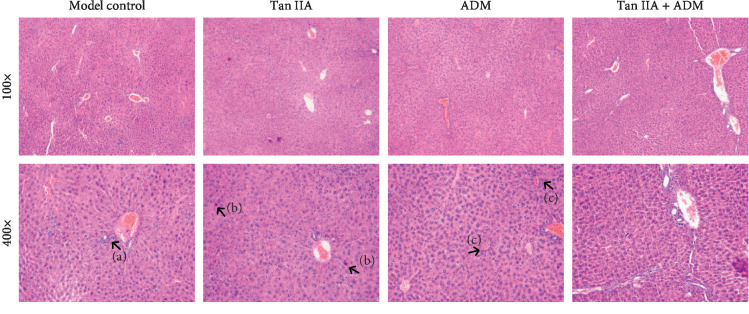
Effects of Tan IIA on liver tissue induced hepatotoxicity in histological appearance by ADM in HepG2 tumor-bearing mice (magnification, ×100 and ×400 as indicated). (a) Inflammatory cells infiltration around the central lobular vein. (b) Local hepatocyte necrosis. (c) Hepatic sinusoid congestion (indicated by arrowheads).

The histopathological changes in liver tissues were examined by H&E staining (Figure 5). Compared with model control group, Tan IIA group congestion was found, with unclear hepatic lobule, inflammatory cells infiltration around the central lobular vein, and few local hepatocyte necrosis. ADM-treated mice displayed severe tissue damage in the liver, including some areas of necrotic hepatocytes, loss of normal crypt architecture, and hepatocyte swelling. In addition, congestion was slightly more visible in the ADM group than in the Tan IIA and combined groups. Congestion and unclear hepatic lobule were found in the Tan IIA group. However, administration of Tan IIA significantly ameliorated ADM-induced liver damage in tumor-bearing mice. Besides, the structure of hepatic lobule in the Tan IIA + ADM group had a lower structural disorder than other groups.

### 3.5. Effects of Cytochrome P450 Contents and Activities in Mice Liver Microsomes

**Figure 6:**
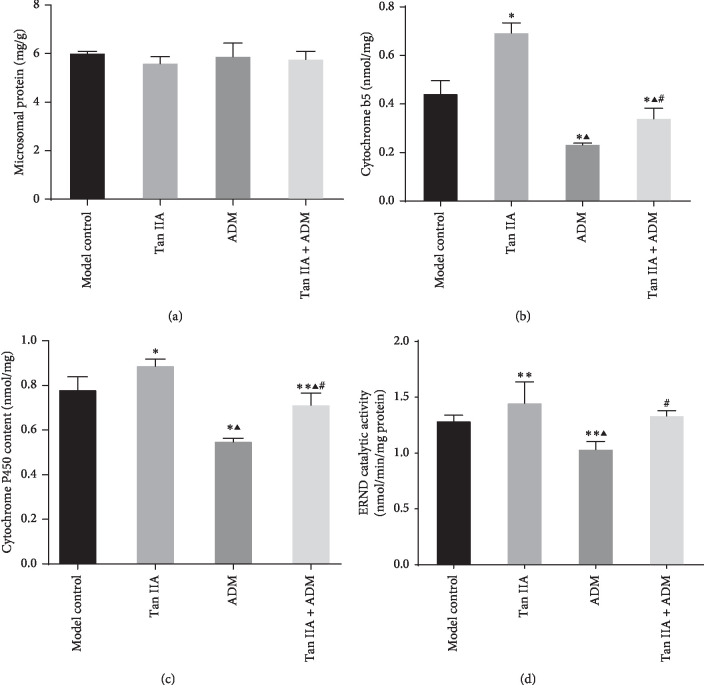
The results of microsomal protein, cytochrome b5, cytochrome P450, and erythromycin-*N*-demethylase (ERND) activity in the liver of HepG2 tumor-bearing mice. (a) The result of liver microsomal protein in tumor-bearing mice. (b) The content of cytochrome b5 in liver microsomes. (c) The content of cytochrome P450 in microsomes. (d) The CYP3A activity in ERND experiment (*n* = 5, ^*∗*^*P* < 0.01, ^*∗∗*^*P* < 0.05 versus control group; ^*▲*^*P* < 0.01 versus TanIIA group; ^#^*P* < 0.01 versus ADM group.)

In the liver of HepG2 tumor-bearing mice, microsome protein content showed no statistical difference (Figure 6(a)). However, cytochrome b5 (Cyt b5), cytochrome P450 content, and erythromycin-*N*-demethylase (ERND) activity showed differences in four groups. The Tan IIA group had the significantly highest content both in Cyt b5 and P450 obviously, and the lowest was in the ADM group. The ratio of cytochrome P450/Cyt b5 was 1.855 : 1, 1.236 : 1, 1.598 : 1, and 1.572 : 1 in four groups, respectively. Compared with control groups, Tan IIA group, ADM group, and combined group all were statistically significant (*P* < 0.01, Figures 6(b) and 6(c)). Compared with the Tan IIA group, both the ADM and combined group remained statistically significant. Among them, the cotreatment of ADM plus Tan IIA group had the statistical significance compared with the ADM group.

The ERND results demonstrated that the CYP3A activity in the Tan IIA treatment group was higher than in the other groups, which had statistical significance compared with the ADM group and the activity result in the ADM group was the lowest. Compared with the model control group, Tan IIA group and ADM group were statistically significant. The cotreatment Tan IIA + ADM showed statistical difference compared with the Tan IIA group, but there was no statistically differences between Tan IIA group and Tan IIA + ADM in ERND results (Figure 6(d)).

### 3.6. Effects of Tan IIA or ADM on CYP450 3A4 Protein Expression in Mice Liver Microsomes

**Figure 7:**
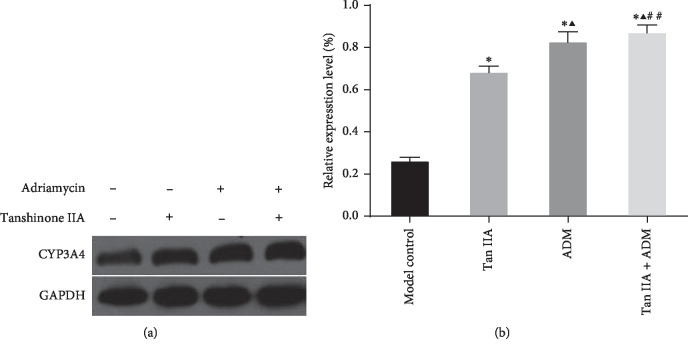
Effect of Tan IIA and ADM, respectively, and jointly on protein expression of CYP3A4 in mice liver microsomes. Asterisks indicate a statistically significant difference compared to the model control group (*n* = 5, ^*∗*^*P* < 0.01 versus control group; ^*▲*^*P* < 0.01 versus TanIIA group; ^#^*P* < 0.01, ^##^*P* < 0.05 versus ADM group).

As shown in Figure 7(a), the CYP3A4 protein expression in the combined group was upregulated than in other groups, indicating that the combination of Tan IIA and ADM may promote the expression of CYP3A4 pathways. The gray values were analyzed by Image Pro-Plus software, which suggested that there was a significant difference in the expression of protein between the combination group and the control group (*P* < 0.01). Compared with the control group, the ADM group, Tan IIA group, and the combination group all had statistical significant differences (*P* < 0.01). Compared with the ADM group, there was a statistical difference in the Tan IIA + ADM group (Figure 7).

### 3.7. Interactions between Tested Drugs and PXR Protein

**Figure 8:**
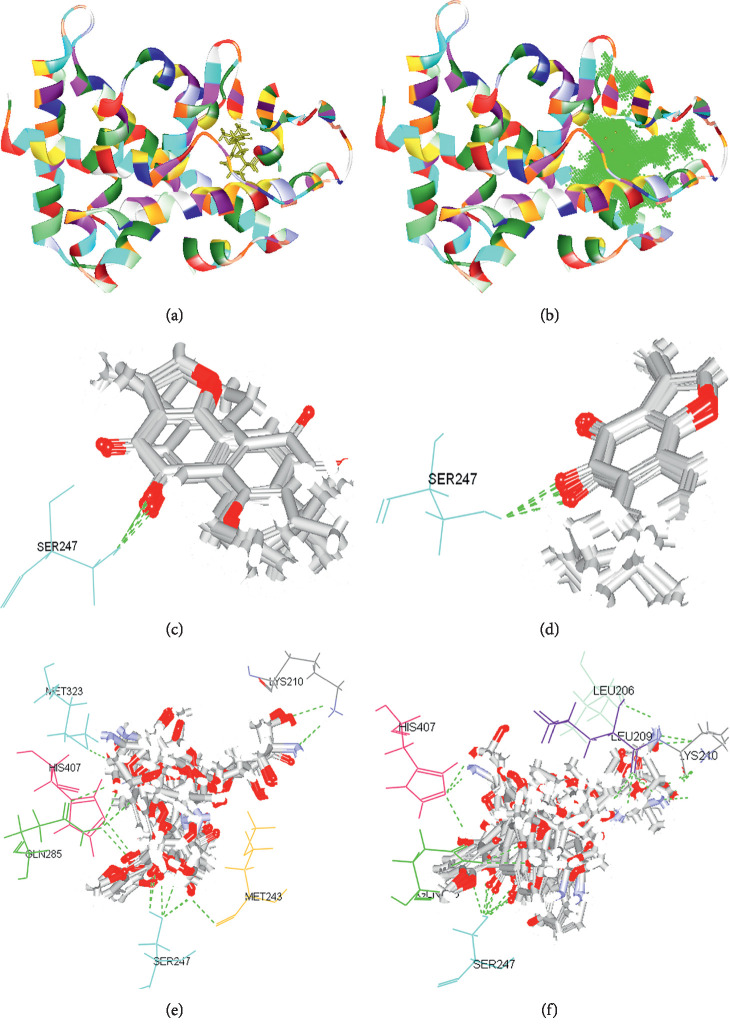
Interactions of Tan IIA and ADM with PXR protein. (a) The 3D structure of PXR from PDB, with an ID of 4NY9 and a resolution of 2.80 Å. It is constituted by 1 peptide chain, which is composed of 290 amino acid residues. An endogenous ligand (yellow) was found within 4NY9. (b) The binding site 1 (green) contains the structure domain of the endogenous ligand after automatic searching. (c) Ten random poses of Tan IIA binding to the endogenous ligand's active site after docking, with one residue involved in the interactions potentially: H-bonds with Ser 247. (d) Ten random poses of Tan IIA binding to site 1 after docking, with the same one residue involved in the interactions potentially: H-bonds with Ser 247. (e) Ten random poses of ADM binding to the endogenous ligand's active site after docking, with six residues involved in the interactions potentially: H-bonds with Lys210, Met243, Ser247, Gln285, Met323, and His407. (f) Ten random poses of ADM binding to site 1 after docking, with five other residues involved in the interactions potentially: H-bonds with Leu206, Leu209, Lys210, Gln285, and His407.

The RMSD of CDOCKER and LibDock was 0.6 Å and 6.7 Å, respectively. Thus, CDOCKER module was selected as the docking method (Table 2). The results showed the resolution ratio of PXR crystal structure from PDB database was 2.8 Å with a peptide chain, consisting of 316 amino acid residues, with endogenous ligand found within the active site of PXR (Figure 8(a)).

After automatic searching, eleven total appropriate active sites were revealed. Among them, site 1 showed a large overlap with the endogenous ligand-binding region. Thus, site 1 and endogenous ligand-binding region were selected as our target binding sites (Figure 8(b)). Second, Tan IIA could be stably docked into two sites with similar interactions, such as the formation of a hydrogen bond with the same residue Ser247 (Figures 8(c) and 8(d)). Third, ADM could also be stably docked into the two sites but with different interactions (Figures 8(e) and 8(f)). In conclusion, we can presume that both Tan IIA and ADM could be stably docked in the internal ligand-binding domain of PXR molecule, but with distinct molecular interactions.

## 4. Discussion

For many years, our research group focused on studying the interaction between active components of herb compounds and traditional anticancer medicine. Now, we are developing an effective platform to evaluate the possible antitumor effect of herbs with chemotherapy drugs to provide experimental evidence and theoretical reference for finding new effective sensitizers. In our previous *in vitro* studies, we explored the mechanisms of action of Tan IIA combined with ADM in many cancer cell lines and found that Tan IIA might suppress A549 proliferation and induce apoptosis and cell cycle arrest at the S phase [10]. We also found that both Tan IIA and ADM could inhibit the growth of A549, PC9, and HLF cells in a dose- and time-dependent manner, while the proliferative inhibition effect of ADM was stronger than of Tan IIA [13].

Therefore, to make the results *in vitro* more effective, we proceeded to study whether the active component of herbs combined with chemotherapeutic drugs could have a synergistic effect in antitumor treatment both *in vitro* and *in vivo* studies. We established the model of human hepatocarcinoma HepG2 xenograft tumor in nude mice to observe the potential anticancer mechanisms of Tan IIA combined with ADM. In preliminary research, we found that the combination of Tan IIA with ADM could decrease the volume of xenograft tumors in mice (data not shown here). In this study, it indicated that Tan IIA might relieve toxicity and other side effects of ADM. Meanwhile, the tumor volumes and weights in the combination group were lower than in other groups. Therefore, we concluded that Tan IIA combined with ADM not only improved the general conditions of tumor-bearing mice but also decreased the tumor volume and increased the antitumor rate.

In this study, we also found that the inhibitory rate of Tan IIA (IR = 32.77%) was lower than that of ADM (IR = 60.96%), indicating the antitumor effects of Tan IIA was weaker than those of ADM. This finding was consistent with other studies [26–30] including our previous study [11]. Although the anticancer activity of the active components was inferior to antineoplastic drugs in herbs, it was widely presumed that the active component responsible for the antitumor effect in herbs has less toxicity in clinical practice [9]. Taking this into consideration, we hypothesized that Tan IIA combined with ADM might work synergistically to result in improving the anticancer effects, as well as reducing the necessary drug dose which may prevent adverse side effects and improve quality of life for patients.

Based on our previous work, our study emphasized the combined effects of Tan IIA and ADM *in vivo*. The results showed that the combination not only affects the tumor volume and inhibitory rate but improves the changes in the appearance of nude mice bearing tumors. These indicate that due to the antitumor efficiency of these two drugs, lower doses of ADM combined with herbs may reduce the chance of side effects. Therefore, as one of the active ingredients from herbs, Tan IIA enhances the treatment of tumors as part of combination therapy and advances our knowledge of using active compounds of the herb as an antitumor ingredient. However, our research studies were still insufficient. Because the method of drug intervention was based on our previous study (data not shown here), the differences in treatment duration between the Tan IIA and the ADM group were unconvincing. Furthermore, it would be more reasonable if there was a drug concentration experiment, like the Tan IIA group having different dose concentrations: low, middle, and high concentrations *in vivo*, even though the medical concentration experiment was involved *in vitro* [13]. So we intend to improve the work during the next stage.

In the past few decades, studies showed that traditional Chinese medicine (TCM) combined with chemotherapy drugs could decrease adverse reactions, overcome multidrug resistance in tumors, improve immune function, and achieve similar or even better curative effects in patients compared to high-dose chemotherapy agents [31]. Tai et al. [32] showed that rosemary extract with the antiproliferation activity of cisplatin showed significant antiproliferation activity on human ovarian cancer A2780. Okamoto et al. [33] showed that cultivation of human peripheral blood mononuclear cells in the presence of cisplatin (0–1.0 *μ*g/mL) or 5-FU (0–5.0 *μ*g/mL) resulted in the significant augmentation of natural killer and lymphokine-activated killer cell activities, as well as generation of interferon-gamma, tumor necrosis factor alpha, beta interleukin (IL)-1 beta, IL-6, and IL-12 *in vitro*. Our results found that Tan IIA not only showed synergistic antitumor effect with ADM but also reduced the toxicity of ADM and improved the quality of life in tumor-bearing mice, which was consistent with our previous assumptions.

It is very common for drugs to be used in combination with clinical practice, especially in cancer management. In China, TCM combined with western medicine is common, which may increase the chances of metabolic drug interactions by drug metabolic enzymes. Therefore, we studied the effects of Tan IIA and ADM with drug metabolic enzymes on human hepatocellular carcinoma xenograft in BALB/c nude mice. Cytochrome P450 enzymes are known as the key enzymes involved in drug metabolism. P450 enzymes are a superfamily, which contains many isoenzymes in liver microsomes. It is documented that there are 57 functional genes and 58 pseudogenes, which metabolize many endogenous substrates such as steroids, eicosanoids, and xenobiotics in P450 enzymes [34–36]. Meanwhile, CYP1, CYP2, and CYP3 are the main subfamily enzymes contributing to the oxidative metabolism of over 95% of clinical drugs, with wide variation observed in hepatic CYP concentration and activity which contributes to *in vivo* differences in drug response [37–40]. The CYP3A subfamily is the most abundant of all CYP isoforms and catalyzes the oxidative metabolism of various clinical drugs. Therefore, it is of fundamental importance to assess the metabolic functions of hepatic CYP3A [41]. In recent years, people found that P450 enzymes participated in a variety of metabolic activations including precarcinogen and protoxin and are closely related to tumor formation. Many *in vitro* studies have shown that Tan IIA and sodium tanshinone IIA sulfonate may inhibit the activities of cytochrome P450 enzyme [42–47], but more of an induction effect rather than inhibition was reported *in vivo* [48–50].

Based on previous P450 enzyme studies [11, 51–54], our results showed that the contents of cytochrome b5 and P450 enzymes and CYP3A activity were decreased in the Tan IIA group. However, there was a significant inhibitory effect in the ADM group, with the Tan IIA plus ADM group close to the level of the control group. The difference was statistically significant, indicating that Tan IIA might be a promising apoptosis inducer to P450 enzymes. Therefore, we speculate that Tan IIA in combination with ADM drugs may increase the content of P450 enzyme and activity in HepG2-bearing mice. The increase may reduce the weak reaction by speeding up the rate of drug metabolism and reducing the acting time of medication in the mouse. This may partly explain why a weak antitumor effect was observed when using Tan IIA alone. ADM, on the other hand, showed an inhibitory effect on xenografts in this study. Researchers had found ADM was a substrate of P450 enzymes and participated in CYP3A metabolic reactions [41, 55, 56]. We found that ADM produced an inhibitory effect on P450 enzymes to some extent, indicating that this inhibition may enhance antitumor activity by increasing the blood concentration of ADM. The high concentration of ADM in blood could be hazardous to mouse health. The decreased appearance of good health of the mice in the ADM group might be related to the inhibitory effect on the P450 enzyme by ADM.

Moreover, our results indicated that the combination of Tan IIA and ADM could induce P450 enzyme activity in tumor-bearing mice. Even more interesting is that we found the ratio of cytochrome P450/b5 was close to 1 : 1 (1.236 : 1) than other groups. Cytochrome b5 was shown to interact with cytochrome P450 to form a tight 1 : 1 complex (Kd = 275 nM), in which the proportion of high-spin cytochrome P450 was increased from 7 to 30% according to recent studies. Increasing P450 enzyme concentration could prevent a rapid decrease in drug concentration due to metabolism, resulting in enhanced antitumor activity, as well as a decrease in toxicity due to the shortening of chemotherapy treatments. Therefore, Tan IIA might be an adjunct medicine to chemotherapy to increase efficacy and reduce toxicity.

CYP3A4 is one of the most important subgroups of cytochrome P450 enzymes and is the predominant CYP expressed in the human liver, accounting for up to 50% of total hepatic CYP protein [57]. It is well known that PXR is a key regulator of a large and growing array of drug targeting genes that correspond to all phases of drug metabolism. Many substances, both endogenous and foreign, activate nuclear receptors involved in CYP3A4 activity, including the pregnane X receptor (PXR), which is a significant regulator of CYP3A4 gene expression. PXR is activated by many ligands and causes the inhibition or induction of drug interaction [58, 59]. Many drugs affect the expression of CYP3A4 by regulating PXR and influencing drug metabolism of the CYP3A4 substrate. Subsequently, PXR was considered to be a key regulator of the CYP3A4 protein [11, 60–62].

Previously, it has been established that the active ingredients of Chinese medicine could activate the PXR/CYP3A4 pathway. For example, the extracts of Wu Wei Zi, Gan Cao, *Echinacea purpurea*, and Byakangelicin could activate PXR to induce the CYP3A4 transcriptional expression in HepG2 cells [63–65]. Other papers have presented convincing evidence that Tan IIA was an efficacious PXR agonist *in vitro*. For example, Yu et al. [60] showed that Dan-Shen ethanol extract could activate human PXR and induce the CYP3A4 reporter construct in HepG2 cells and identified Tan IIA as an efficacious PXR agonist. Zhang et al. [66] revealed that Tan IIA could protect against LCA-induced hepatotoxicity and cholestasis in a dose-dependent manner in *in vivo* experiments using PXR siRNA. Zhang et al. [62] demonstrated that Tan IIA is an efficacious PXR agonist and able to increase CYP3A4 mRNA levels, and its protein expression was mediated by the transactivation of PXR in inflammatory bowel disease. These studies suggested that PXR might play an important role in the relationship of Tan IIA and CYP3A4.

Previously, it has been shown that PXR mRNA expression in LS174T cells was significantly induced by physcion, protocatechuic aldehyde, salvianolic acid B, and sodium Dan-Shen Su. However, Tan IIA significantly downregulated the expression of PXR mRNA in LS174T cells, suggesting that herbal medicines could significantly regulate PXR and CYP3A4, which has an important implication in herb-drug interactions [11]. Our results showed the expression of CYP3A4 protein was upregulated significantly in the combination group. Our member also found that Tan IIA could induce the expression of recombinant proteins of PXR in HepG2 tumor-bearing mice (data not shown). Then, we hypothesize that Tan IIA, as one of the efficacious PXR agonists, might enhance the ADM substrate in combination with PXR and improve the protein expression of CYP3A4 in mice bearing HepG2 cell xenograft tumors.

PXR is a key regulator of a large and growing array of drug-targeting genes that correspond to CYP3A4 liver enzymes. It has a large and flexible ligand-binding domain (LBD), allowing PXR to be activated by a wide variety of compounds. Our previous study showed that Tan IIA and other herbal compounds tested can be readily docked into the ligand-binding cavity of PXR mainly through hydrogen bonds and aromatic interactions with Ser247, Gln285, His407, and Arg401 [11]. However, the study and application on the virtual docking of ADM with the PXR protein have not been reported. Considering the research mentioned previously, we speculate that Tan IIA combined with ADM might act as an activator of PXR, forming complexes with PXR to induce the production of the downstream protein CYP3A4, subsequently inhibiting tumor progression.

Considering our previous studies [11, 20, 51, 52], we explored the interactions of traditional Chinese herbal formulas with the human cytochrome P450 by virtual screening. We also explored interactions with Tan IIA. Xie et al. [10] found that Tan IIA could be stably docked into the kinase domain of VEGFR2 protein to form hydrogen bonds with Cys917 and *π*–*π* stacking interactions with Val848. Xie et al. speculated that Tan IIA might act as a competitive inhibitor of VEGFR2, subsequently inhibiting tumor angiogenesis, cell migration, and tumorigenicity. In further research, Xie et al. [13] found Tan IIA could be docked into the active sites of all tested proteins with hydrogen bonds and aromatic interactions, compared with that of ADM.

In the final stages of our study, we simulated the interactions of targeted drugs with PXR protein by the virtual docking research platform. After the method was optimized using the results of molecular biological experiments, we focused on the analysis of possible binding models of Tan IIA and ADM. In the PDB database, our molecular docking analysis results found the three-dimensional structure of PXR (PDB-ID : 4NY9), including the PXR protein kinase structural domain (resolution ratio of 2.80 Å) by automatic searching using DS 2.1. We found that site 1 can cover the structural domain included by endogenous ligand. After molecule docking in two ways, both Tan IIA and ADM could target the protein kinase domains of PXR. In the domain, Tan IIA is an effective PXR agonist, so we concluded that the docking site of Tan IIA and PXR may be defined as the structural domain of the binding site for endogenous ligand. In the kinase structural domain, ADM docking with PXR endogenous ligands was higher than Tan IIA, indicating that ADM and PXR may have more stable docking. Moreover, there is a complementarity at the interaction/binding sites in molecular docking, which has been confirmed by recent study [13]. Our findings suggest that the structure of these docking sites may have influence on the synergistic antitumor effects, and further study is needed.

Our investigation indicates that Tan IIA in combination with ADM is a promising effective cancer therapy treatment. This new potential combination therapy needs to be further developed, as it could minimize adverse side effect to significantly improve the quality of life of cancer patients.

## 5. Conclusions

This study found that Tan IIA in combination with ADM could improve the quality of life of tumor-bearing mice and enhance the antitumor effect. The Tan IIA increases the concentration of cytochrome P450 enzymes and activity. Combination of Tan IIA and ADM could upregulate the CYP3A4 protein expression. Both Tan IIA and ADM had a stable virtual docking with the same binding sites with PXR protein. Our study built a feasible platform to evaluate the combinatorial antitumor effects of herbs and chemotherapy drugs in an *in vivo* study.

## Figures and Tables

**Table 1:** The inhibitory rate following treatment in four groups.

Groups	Inhibitory rate (%)
Model control	—
Tan IIA	32.77
ADM	60.96
Tan IIA + ADM	73.18

**Table 2:** The RMSD of two modules after redock (Å).

Protein	CDOCKER RMSD	LibDock RMSD
4NY9	0.6	6.7

## Data Availability

The data used to support the findings of this study are available from the corresponding authors (Xin-Lin Wu and Sui-Lin Mo) upon request.

## References

[1] Torre L. A., Bray F., Siegel R. L., Ferlay J., Lortet-Tieulent J., Jemal A. (2015). Global cancer statistics, 2012. *CA: A Cancer Journal for Clinicians*.

[2] Ferlay J., Soerjomataram I., Dikshit R. (2015). Cancer incidence and mortality worldwide: sources, methods and major patterns in GLOBOCAN 2012. *International Journal of Cancer*.

[3] Yamamoto S., Kondo S. (2018). Oral chemotherapy for the treatment of hepatocellular carcinoma. *Expert Opinion on Pharmacotherapy*.

[4] Song Y., Jang J., Shin T. H. (2017). Sulfasalazine attenuates evading anticancer response of CD133-positive hepatocellular carcinoma cells. *Journal of Experimental & Clinical Cancer Research*.

[5] Liu M. L., Chien L. Y., Tai C. J. (2011). Effectiveness of traditional Chinese medicine for liver protection and chemotherapy completion among cancer patients. *Evidence-Based Complementary and Alternative Medicine*.

[6] Jiang L., Huang L., Kuang Q. (2014). Improving the prediction of chemotherapeutic sensitivity of tumors in breast cancer via optimizing the selection of candidate genes. *Computational Biology and Chemistry*.

[7] Li F., Zhang W. (2014). Role of traditional Chinese medicine and its chemical components in anti-tumor metastasis. *Journal of Cancer Research and Therapeutics*.

[8] Ling B., Michel D., Sakharkar M., Yang J. (2016). Evaluating the cytotoxic effects of the water extracts of four anticancer herbs against human malignant melanoma cells. *Drug Design, Development and Therapy*.

[9] Hsiao W., Liu L. (2010). The role of traditional Chinese herbal medicines in cancer therapy—from TCM theory to mechanistic insights. *Planta Medica*.

[10] Xie J., Liu J., Liu H. (2015). The antitumor effect of tanshinone IIA on anti-proliferation and decreasing VEGF/VEGFR2 expression on the human non-small cell lung cancer A549 cell line. *Acta Pharmaceutica Sinica B*.

[11] Liu Y.-H., Mo S.-L., Bi H.-C. (2011). Regulation of human pregnane X receptor and its target gene cytochrome P450 3A4 by Chinese herbal compounds and a molecular docking study. *Xenobiotica*.

[12] Thorn C. F., Oshiro C., Marsh S. (2011). Doxorubicin pathways. *Pharmacogenetics and Genomics*.

[13] Xie J., Liu J. H., Liu H. (2016). Tanshinone IIA combined with adriamycin inhibited malignant biological behaviors of NSCLC A549 cell line in a synergistic way. *BMC Cancer*.

[14] Munagala R., Aqil F., Jeyabalan J., Gupta R. C. (2015). Tanshinone IIA inhibits viral oncogene expression leading to apoptosis and inhibition of cervical cancer. *Cancer Letters*.

[15] Chiu S. C., Huang S. Y., Chen S. P., Su C. C., Chiu T. L., Pang C. Y. (2013). Tanshinone IIA inhibits human prostate cancer cells growth by induction of endoplasmic reticulum stress *in vitro* and *in vivo*. *Prostate Cancer and Prostatic Diseases*.

[16] Yang L., Guo H., Dong L., Wang L., Liu C., Wang X. (2014). Tanshinone IIA inhibits the growth, attenuates the stemness and induces the apoptosis of human glioma stem cells. *Oncology Reports*.

[17] Wang W.-Q., Liu L., Sun H.-C. (2012). Tanshinone IIA inhibits metastasis after palliative resection of hepatocellular carcinoma and prolongs survival in part via vascular normalization. *Journal of Hematology & Oncology*.

[18] Hong H.-J., Liu J.-C., Chen P.-Y., Chen J.-J., Chan P., Cheng T.-H. (2012). Tanshinone IIA prevents doxorubicin-induced cardiomyocyte apoptosis through Akt-dependent pathway. *International Journal of Cardiology*.

[19] Gao J., Yang G., Pi R. (2008). Tanshinone IIA protects neonatal rat cardiomyocytes from adriamycin-induced apoptosis. *Translational Research*.

[20] Gu Y.-Y., Liu L.-P., Qin J. (2014). Baicalein decreases side population proportion via inhibition of ABCG2 in multiple myeloma cell line RPMI 8226 *in vitro*. *Fitoterapia*.

[21] Zhu H. Y., Cao G. Y., Wang S. P. (2017). POU2F1 promotes growth and metastasis of hepatocellular carcinoma through the FAT1 signaling pathway. *American Journal of Cancer Research*.

[22] Deng Y., Bi H.-C., Zhao L.-Z. (2008). Induction of cytochrome P450s by terpene trilactones and flavonoids of theGinkgo bilobaextract EGb 761 in rats. *Xenobiotica*.

[23] Lowry O. H., Rosebrough N. J., Farr A. L., Randall R. J. (1951). Protein measurement with the Folin phenol reagent. *The Journal of Biological Chemistry*.

[24] Tu Y. Y., Yang C. S. (1983). High-affinity nitrosamine dealkylase system in rat liver microsomes and its induction by fasting. *Cancer Research*.

[25] Fu G., Zhou C., Wang Y. (2016). Effects of inducers of cytochrome P450s on enrofloxacin N -deethylation in crucian carp *Carassius auratus* gibelio. *Environmental Toxicology and Pharmacology*.

[26] Zhang J., Wang J., Jiang J.-Y., Liu S.-D., Fu K., Liu H.-Y. (2014). Tanshinone IIA induces cytochrome c-mediated caspase cascade apoptosis in A549 human lung cancer cells via the JNK pathway. *International Journal of Oncology*.

[27] Kan S., Cheung W., Zhou Y., Ho W. (2014). Enhancement of doxorubicin cytotoxicity by tanshinone IIA in HepG2 human hepatoma cells. *Planta Medica*.

[28] Chan S. E., Lai H. W., Su C. C. (2011). Effect of supplementation of tanshinone IIA and sodium tanshinone IIA sulfonate on the anticancer effect of epirubicin: an *in vitro* study. *Evidence-Based Complementary and Alternative Medicine*.

[29] Liu X., Wang Y., Ma C. (2011). Proteomic assessment of tanshinone II A sodium sulfonate on doxorubicin induced nephropathy. *The American Journal of Chinese Medicine*.

[30] Wang X., Wei Y., Yuan S. (2005). Potential anticancer activity of tanshinone IIA against human breast cancer. *International Journal of Cancer*.

[31] Emadi A., Jones R. J., Brodsky R. A. (2009). Cyclophosphamide and cancer: golden anniversary. *Nature Reviews Clinical Oncology*.

[32] Tai J., Cheung S., Wu M., Hasman D. (2012). Antiproliferation effect of Rosemary (*Rosmarinus officinalis*) on human ovarian cancer cells *in vitro*. *Phytomedicine*.

[33] Okamoto M., Kaji R., Goda H. (Dec 1998). Cis -Diamminedichloroplatinum and 5-fluorouracil are potent inducers of the cytokines and natural killer cell activity in vivo and in vitro. *Cancer Immunology, Immunotherapy*.

[34] Nebert D. W., Russell D. W. (2002). Clinical importance of the cytochromes P450. *Lancet*.

[35] Jiang B., Zhang L., Wang Y. (2009). Tanshinone IIA sodium sulfonate protects against cardiotoxicity induced by doxorubicin *in vitro* and *in vivo*. *Food and Chemical Toxicology*.

[36] Wang R. W., Newton D. J., Liu N., Atkins W. M., Lu A. Y. (2000). Human cytochrome p-450 3a4 in vitro drug-drug interaction patterns are substrate-dependent. *Drug Metabolism and Disposition*.

[37] Ingelman-Sundberg M., Sim S. C., Gomez A., Rodriguez-Antona C. (2007). Influence of cytochrome P450 polymorphisms on drug therapies: pharmacogenetic, pharmacoepigenetic and clinical aspects. *Pharmacology & Therapeutics*.

[38] Zhou S.-F., Liu J.-P., Chowbay B. (2009). Polymorphism of human cytochrome P450 enzymes and its clinical impact. *Drug Metabolism Reviews*.

[39] Roberts B. J., Shoaf S. E., Song B. J. (1995). Rapid changes in cytochrome P4502E1 (CYP2E1) activity and other P450 isozymes following ethanol withdrawal in rats. *Biochemical Pharmacology*.

[40] Mimura N., Kobayashi K., Nakamura Y., Shimada N., Hosokawa M., Chiba K. (2003). Metabolism of medroxyprogesterone acetate (MPA) via CYP enzymes *in vitro* and effect of MPA on bleeding time in female rats in dependence on CYP activity *in vivo*. *Life Sciences*.

[41] Nagai K., Yoshida N., Kiyama M., Kasahara K., Yamamura A., Konishi H. (2015). Decreased elimination clearance of midazolam by doxorubicin through reductions in the metabolic activity of hepatic CYP3A in rats. *Xenobiotica*.

[42] Ouyang D. S., Huang W. H., Chen D. (2016). Kinetics of cytochrome P450 enzymes for metabolism of sodium tanshinone IIA sulfonate *in vitro*. *Chinese Medicine*.

[43] Chen D., Lin X. X., Huang W. H. (2016). Sodium tanshinone IIA sulfonate and its interactions with human CYP450s. *Xenobiotica; the fate of foreign compounds in biological systems*. *Mar*.

[44] He F., Bi H.-c., Xie Z.-y. (2007). Rapid determination of six metabolites from multiple cytochrome P450 probe substrates in human liver microsome by liquid chromatography/mass spectrometry: application to high-throughput inhibition screening of terpenoids. *Rapid Communications in Mass Spectrometry*.

[45] Jeon Y. J., Kim J. S., Hwang G. H. (2015). Inhibition of cytochrome P450 2J2 by tanshinone IIA induces apoptotic cell death in hepatocellular carcinoma HepG2 cells. *European Journal of Pharmacology*.

[46] Qiu F., Zhang R., Sun J. (2008). Inhibitory effects of seven components of danshen extract on catalytic activity of cytochrome P450 enzyme in human liver microsomes. *Drug Metabolism and Disposition*.

[47] Wang R., Zhang H., Wang Y., Yu X., Yuan Y. (2016). Effects of salvianolic acid B and tanshinone IIA on the pharmacokinetics of losartan in rats by regulating the activities and expression of CYP3A4 and CYP2C9. *Journal of Ethnopharmacology*.

[48] Ueng Y.-F., Kuo Y.-H., Wang S.-Y., Lin Y.-L., Chen C.-F. (2004). Induction of CYP1A by a diterpene quinone tanshinone IIA isolated from a medicinal herb Salvia miltiorrhiza in C57BL/6J but not in DBA/2J mice. *Life Sciences*.

[49] Kuo Y. H., Lin Y. L., Don M. J., Chen R. M, Ueng Y. F (2006). Induction of cytochrome P450-dependent monooxygenase by extracts of the medicinal herb Salvia miltiorrhiza. *The Journal of Pharmacy and Pharmacology*.

[50] Chen Y., Tu J.-H., He Y.-J. (2009). Effect of sodium tanshinone II A sulfonate on the activity of CYP1A2 in healthy volunteers. *Xenobiotica*.

[51] Mo S.-L., Liu W.-F., Chen Y. (2012). Ligand- and protein-based modeling studies of the inhibitors of human cytochrome P450 2D6 and a virtual screening for potential inhibitors from the Chinese Herbal Medicine, *Scutellaria baicalensis* (Huangqin, Baikal Skullcap). *Combinatorial Chemistry & High Throughput Screening*.

[52] Mo S.-L., Liu W.-F., Li C.-G. (2012). Pharmacophore, QSAR, and binding mode studies of substrates of human cytochrome P450 2D6 (CYP2D6) using molecular docking and virtual mutations and an application to Chinese herbal medicine screening. *Current Pharmaceutical Biotechnology*.

[53] Mo S.-L., Liu Y.-H., Duan W., Wei M., Kanwar J., Zhou S.-F. (2009). Substrate specificity, regulation, and polymorphism of human cytochrome P450 2B6. *Current Drug Metabolism*.

[54] Mo S.-L., Zhou Z.-W., Yang L.-P., Wei M., Zhou S.-F. (2009). New insights into the structural features and functional relevance of human cytochrome P450 2C9. Part I. *Current Drug Metabolism*.

[55] Lu H., Chen C.-S., Waxman D. J. (2009). Potentiation of methoxymorpholinyl doxorubicin antitumor activity by P450 3A4 gene transfer. *Cancer Gene Therapy*.

[56] Choi J. S., Piao Y. J., Kang K. W. (2011). Effects of quercetin on the bioavailability of doxorubicin in rats: role of CYP3A4 and P-gp inhibition by quercetin. *Archives of Pharmacal Research*.

[57] Wacher V. J., Wu C.-Y., Benet L. Z. (1995). Overlapping substrate specificities and tissue distribution of cytochrome P450 3A and P-glycoprotein: implications for drug delivery and activity in cancer chemotherapy. *Molecular Carcinogenesis*.

[58] Betts S., Björkhem-Bergman L., Rane A., Ekström L. (2015). Expression of CYP3A4 and CYP3A7 in human foetal tissues and its correlation with nuclear receptors. *Basic &amp; Clinical Pharmacology &amp; Toxicology*.

[59] Wei P., Zhang J., Dowhan D. H., Han Y., Moore D. D. (2002). Specific and overlapping functions of the nuclear hormone receptors CAR and PXR in xenobiotic response. *The Pharmacogenomics Journal*.

[60] Yu C., Ye S., Sun H. (2009). PXR-mediated transcriptional activation of CYP3A4 by cryptotanshinone and tanshinone IIA. *Chemico-biological Interactions*.

[61] Holmstock N., Gonzalez F. J., Baes M., Annaert P., Augustijns P. (2013). PXR/CYP3A4-humanized mice for studying drug-drug interactions involving intestinal P-glycoprotein. *Molecular Pharmaceutics*.

[62] Zhang X., gao Y., wang Y. (2015). Tanshinone IIA ameliorates dextran sulfate sodium-induced inflammatory bowel disease via the pregnane X receptor. *Drug Design, Development and Therapy*.

[63] Mu Y., Zhang J., Zhang S. (2006). Traditional Chinese medicines Wu Wei Zi (Schisandra chinensis Baill) and Gan Cao (*Glycyrrhiza uralensis* fisch) activate pregnane X receptor and increase warfarin clearance in rats. *Journal of Pharmacology and Experimental Therapeutics*.

[64] Awortwe C., Manda V. K., Avonto C. (2015). Echinacea purpureaup-regulates CYP1A2, CYP3A4 and MDR1 gene expression by activation of pregnane X receptor pathway. *Xenobiotica*.

[65] Yang J., Luan X., Gui H. (2011). Byakangelicin induces cytochrome P450 3A4 expression via transactivation of pregnane X receptors in human hepatocytes. *British Journal of Pharmacology*.

[66] Zhang X., Ma Z., Liang Q. (2015). Tanshinone IIA exerts protective effects in a LCA-induced cholestatic liver model associated with participation of pregnane X receptor. *Journal of Ethnopharmacology*.

